# Intervention Potential of a Recombinant Tarim Red Deer HGF Protein in a Mouse Model of Alcoholic Liver Disease

**DOI:** 10.3390/biology14070790

**Published:** 2025-06-30

**Authors:** Hong Chen, Chuan Lin, Xin Xiang, Chenchen Yang, Chunmei Han, Qinghua Gao

**Affiliations:** 1 College of Life Science and Technology, Tarim University, Alar 843300, China; 107572024416@stumail.taru.edu.cn; 2 College of Animal Science and Technology, Tarim University, Alar 843300, China; linchuan0528@163.com (C.L.); 10757223056@stumail.taru.edu.cn (X.X.); 10757223066@stumail.taru.edu.cn (C.Y.); 3 Key Laboratory of Tarim Animal Husbandry Science and Technology, Xinjiang Production and Construction Corps, Alar 843300, China; 4 Key Laboratory of Livestock and Forage Resources Utilization Around Tarim, Ministry of Agriculture and Rural Areas, Alar 843300, China

**Keywords:** alcoholic liver disease, recombinant HGF protein, regenerative therapy, HGF/c-Met pathway

## Abstract

This study investigates the recombinant Tarim red deer hepatocyte growth factor (HGF) in a mouse model to develop an HGF/c-Met-based regenerative therapy for alcoholic liver disease. We constructed a recombinant HGF fusion protein and expressed and purified it in *Escherichia coli*. The recombinant protein was administered via intravenous injection to treat mice with alcoholic liver disease induced by chronic alcohol feeding followed by acute alcohol gavage (NIAAA model). The therapeutic effects were evaluated based on liver tissue histology and biochemical indicators. The recombinant Tarim red deer HGF protein successfully reduced serum aspartate aminotransferase (AST) and alanine aminotransferase (ALT) levels in mice, increased serum albumin (ALB) levels, decreased hepatic steatosis and triglyceride (TG) levels, lowered hepatic malondialdehyde (MDA) levels, and increased the levels of the antioxidants glutathione (GSH) and superoxide dismutase (SOD) in the liver. Additionally, it enhanced the proliferation capacity of liver cells, thereby promoting liver regeneration. In conclusion, our study demonstrates that recombinant Tarim red deer HGF effectively reduces liver damage in a mouse model of alcoholic liver disease.

## Simple Summary

Alcoholic liver disease represents a significant health concern resulting from excessive alcohol consumption, impacting millions of individuals worldwide and leading to liver damage, inflammation, and even cancer. Current treatment options are limited, underscoring the necessity for novel therapeutic strategies. This study explores the potential of a recombinant Tarim red deer hepatocyte growth factor (HGF) protein as a regenerative therapy for alcoholic liver disease. The research involved the construction of a fusion protein derived from the HGF of Tarim red deer and the assessment of its effects in a mouse model of alcoholic liver disease. The findings indicate that the recombinant HGF protein markedly reduced liver damage by decreasing levels of liver injury markers, minimizing liver fat deposition, and enhancing liver cell regeneration. This study highlights the therapeutic potential of recombinant Tarim red deer HGF in the treatment of alcoholic liver disease, paving the way for future research and clinical applications.

## 1. Introduction

As the primary site of alcohol metabolism, the liver is the organ most commonly affected by alcohol. The substantial amount of toxic metabolites produced during alcohol metabolism in the liver can lead to the development of alcoholic liver disease (ALD) [1]. Clinical liver diseases resulting from excessive alcohol consumption, collectively referred to as ALD, encompass a spectrum of conditions characterized by liver damage, inflammation, liver fibrosis, cirrhosis, and cancer [2]. According to the 2018 Global Alcohol and Health Report published by the World Health Organization, approximately 3 million people die each year worldwide due to excessive alcohol consumption, representing 5.3% of all annual deaths. Among those who succumbed to cirrhosis, 47.9% of cases were attributed to excessive alcohol consumption [2]. The International Agency for Research on Cancer (IARC) reports that approximately 740,000 new cases of cancer globally can be linked to alcohol consumption, with esophageal and liver cancers being the two most prevalent types [3]. Liver cancer is estimated to account for about 154,700 new cases, or 20.9% of the total number of new cancer cases diagnosed each year [3]. Currently, there are no specific drugs approved for the treatment of ALD.

Hepatocyte growth factor/scatter factor (HGF/SF) is primarily synthesized and secreted by mesenchymal cells, activating c-Met receptors that mediate the recruitment of downstream adapter proteins and direct kinases. This process transduces signals to downstream pathways, including JAK/STAT3, PI3K/Akt/NFκB, Ras/Raf, and MAPK pathways, which influence cell motility, proliferation, invasion, survival, morphogenesis, and angiogenesis [4,5,6]. Due to its significant biological activity, HGF has garnered considerable attention in regenerative therapy and has been applied in the treatment of various diseases, wound healing, and tissue regeneration [7,8]. Wang et al. demonstrated that mesenchymal stem cells secrete HGF to mitigate acetaminophen-induced liver toxicity [9]. Additionally, Li et al. found that the injection of recombinant human HGF promotes the phosphorylation of JAK2-STAT3 in mouse liver and inhibits inflammation, thereby enhancing liver function in mice with non-alcoholic fatty liver disease [10]. These studies highlight the crucial role of the HGF/c-Met pathway in liver regeneration and underscore the significant implications for developing candidate drugs targeting this pathway for the treatment of alcoholic liver disease.

Due to the large size of HGF, with a molecular weight of approximately 80 kDa, and the challenges associated with its post-translational modifications, the production hosts are limited to insect or mammalian cells. This constraint renders the production of HGF complex, costly, and significantly restricts its applications. To address this limitation, Nola et al. developed a method for producing a minimal HGF/SF Receptor (MET) agonist in large quantities using Escherichia coli [11]. To assess the therapeutic efficacy of the recombinant Tarim red deer HGF/SF Receptor (MET) agonist produced via this method, we established a mouse model of alcoholic liver disease characterized by chronic alcohol feeding followed by acute alcohol gavage, referred to as the NIAAA or Gao-Binge model [12]. This model more accurately reflects acute human hepatitis [13]. The recombinant Tarim red deer HGF protein obtained was utilized for treatment, and its therapeutic effects on mouse alcoholic liver disease were evaluated by measuring liver histology, blood biochemical indicators, and liver biochemical markers.

## 2. Materials and Methods

### 2.1. Ethics Statement

The animal study protocol was approved by the Technology Ethics Committee of Tarim University (Approval Number: PA2025312084; date of approval: 12 March 2025).

### 2.2. RNA Extraction and cDNA Synthesis

The Tarim red deer antler was ground into a powder using liquid nitrogen. RNA was extracted using Trizol (Invitrogen, Carlsbad, CA, USA) reagent, and subsequently, the RNA was reverse transcribed into cDNA using HyperScript III RT SuperMix with gDNA Remover Kit (EnzyArtisan, Shanghai, China) according to the manufacturer’s protocol. The cDNA was stored at −20 °C.

### 2.3. Construction of Prokaryotic Expression Vector

Based on the Tarim red deer HGF gene sequence obtained from previous experiments [14], two pairs of PCR primers were designed to amplify the Kringle1 domain. Vector homologous sequences were added to both sides of the primers, and a pair of primers was designed to construct the linearized pET45b plasmid (Novagen, North Bethesda, MA, USA). The primers are listed in Table 1. PCR was performed using TransStart^®^ FastPfu Fly DNA Polymerase high-fidelity enzyme (Transgen, Beijing, China). The PCR products were purified using a universal DNA purification recovery kit (Tiangen, Beijing, China).

**Figure 1:**
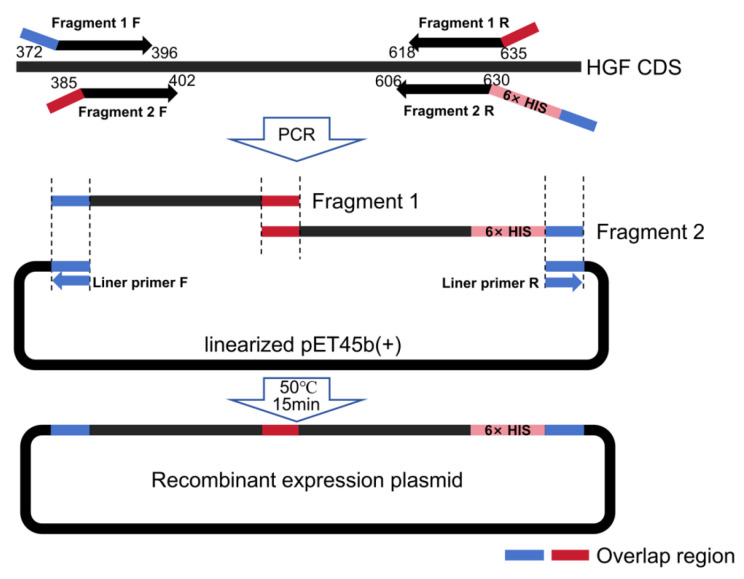
Schematic diagram of constructing expression vector for recombinant Tarim red deer HGF/SF Receptor (MET) agonist.

The fragments 1 and 2, along with the pET45b plasmid, were recombined using the Seamless Cloning Kit (Beyotime, Shanghai, China). The construction scheme for the recombinant expression plasmid is illustrated in Figure 1. The ligated product was transformed into BL21(DE3) Escherichia coli competent cells (Transgen, Beijing, China). The presence of the insert in the plasmid was confirmed through DNA sequencing.

### 2.4. Expression and Purification of Recombinant Tarim Red Deer HGF

The engineered recombinant Tarim red deer hepatocyte growth factor (HGF) was produced as described by Nola et al. [11]. In summary, the recombinant Tarim red deer HGF was expressed in inclusion bodies using BL21 (DE3) *E. coli*. The protein was extracted from these inclusion bodies with a Tris-buffered solution containing 2 M arginine, subsequently diluted in Tris buffer at pH 7.4, and purified using Ni-NTA His Bind Resin (7sea, Shanghai, China). The protein expression and purification were analyzed via SDS-PAGE and quantified using the BCA assay (Beyotime, Shanghai, China).

### 2.5. Animal Experiments

In accordance with the mouse model of alcoholic liver disease established by Bertola et al. [12], this study selected male C57BL/6 mice aged 8 to 10 weeks, weighing 21 ± 1 g (purchased from Takara Bio, Shiga, Japan), which were housed at the Animal Science Experimental Base of Tarim University. The mice were maintained under a 12-h light/dark cycle and were provided with standard rodent chow and free access to water. The experiment was divided into a healthy control group (6 mice) and an alcohol-induced liver injury group (24 mice) to establish the NIAAA mouse model of alcoholic liver disease.

**Figure 2:**
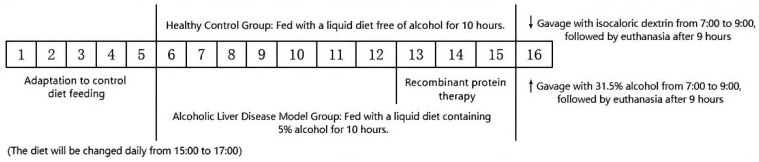
The experimental procedure of chronic alcohol feeding combined with acute alcohol gavage.

On the 13th day of modeling, the mice in the modeling group were randomly divided into four groups (*n* = 6 per group): the alcoholic liver disease model group, the 2 μg recombinant protein treatment group, the 10 μg recombinant protein treatment group, and the 50 μg recombinant protein treatment group. On the 13th, 14th, and 15th days of modeling, the mice in the control group and the alcohol-induced liver disease model group received intravenous injections of sterile physiological saline (250 μL). In contrast, the recombinant protein treatment groups were administered 2 μg, 10 μg, and 50 μg of recombinant protein, respectively, also in a volume of 250 μL. The mouse experimental procedure is illustrated in Figure 2.

On the morning of the 16th day, the mice in the alcohol-induced liver injury model were administered 31.5% ethanol via gavage. After 9 h, the surviving mice were weighed under isoflurane anesthesia. Blood samples were collected and centrifuged at 4 °C and 1000× *g* for 10 min. The serum was then transferred to a new centrifuge tube and stored at −80 °C. Following blood collection, the mouse abdominal wall was dissected using sterile surgical scissors, and the liver was bluntly separated with a sterile cotton swab. The liver was washed with saline and weighed. Subsequently, the liver was divided into five pieces: one piece was placed in a centrifuge tube containing 4% paraformaldehyde, one piece was embedded in OCT at −80 °C, and the remaining three pieces were aliquoted into centrifuge tubes and briefly stored in liquid nitrogen before being transferred to a −80 °C freezer for storage.

### 2.6. Histologic Examination

#### 2.6.1. H&E Staining of Liver

The liver, fixed overnight in 4% paraformaldehyde, underwent dehydration and embedding in paraffin. Subsequently, the embedded liver was sectioned into 6 μm slices, followed by deparaffinization, rehydration, and staining with hematoxylin and eosin. The sections were then observed and photographed under a microscope.

#### 2.6.2. Oil Red O Staining of Liver

After cutting the sample in OCT into 10 μm thick sections, the sections were allowed to sit at room temperature for 20 min. They were then fixed in 20% formalin for 30 min and subsequently washed twice with distilled water for 3 min each. Following this, the sections were immersed in 60% isopropanol for 20 s and stained with oil red O solution for 10 min. After staining, the sections were differentiated in 60% isopropanol for 5 s and rinsed with running water for 1 min. Subsequently, the sections were stained with hematoxylin solution for 1 min and treated with 1% hydrochloric acid in ethanol for 10 s. After a 10 min rinse in running water, the sections were mounted using glycerol jelly mounting medium. The sections were then observed and photographed under a microscope.

#### 2.6.3. Blood and Liver Biochemistry Tests

The assay kits produced by the Nanjing Jiancheng Bioengineering Institute (Nanjing, China) were utilized to detect blood biochemical indicators, including ALT, AST, and ALB levels, as well as liver biochemical indicators such as TG, SOD, GSH, and MDA levels.

### 2.7. Immunofluorescence Detection of Cell Proliferation Marker (Proliferating Cell Nuclear Antigen, PCNA)

Immunofluorescence staining was conducted to evaluate the impact of recombinant Tarim red deer HGF protein on hepatic cell proliferation in a mouse model of alcoholic liver disease. Frozen sections, 6 μm in thickness, were thawed at room temperature for 20 min and subsequently fixed with pre-cooled methanol at −20 °C for 10 min. The sections were washed three times with PBS buffer and then blocked with a blocking buffer (prepared by adding 0.5 mL of goat serum and 30 µL of Triton™ X-100 to 9.5 mL of 1× PBS) for 1 h. Mouse anti-PCNA Monoclonal Antibody (Proteintech, Wuhan, China) was diluted 1:250 in an antibody dilution buffer (prepared by adding 0.1 g BSA and 30 µL Triton™ X-100 to 10 mL of 1× PBS) and incubated overnight at 4 °C, followed by three washes with PBS for 5 min each. The Cy3-conjugated goat anti-mouse secondary antibody (Boster, Wuhan, China) was diluted 1:100 in the antibody dilution buffer and incubated for 1 h in the dark. The sections were then washed three times with PBS for 5 min each. DAPI was added to the sections for 10 min at room temperature for nuclear staining. The sections were washed three times with PBS for 5 min each and then mounted with antifading mounting medium (Solarbio, Beijing, China). Finally, the sections were observed and photographed under a microscope.

### 2.8. Statistical Method

Data analysis was conducted using SPSS version 24.0. The results are presented as means ± standard deviation (SD). One-way ANOVA was employed to assess differences in the experimental data, followed by post hoc testing using the least significant difference (LSD) method. The notation ‘ns’ indicates *p* > 0.05, signifying no significant difference; ‘*’ indicates *p* < 0.05, denoting a significant difference; and ‘**’ indicates *p* < 0.01, reflecting an extremely significant difference.

## 3. Results

### 3.1. Expression and Purification of Recombinant Tarim Red Deer HGF

**Figure 3:**
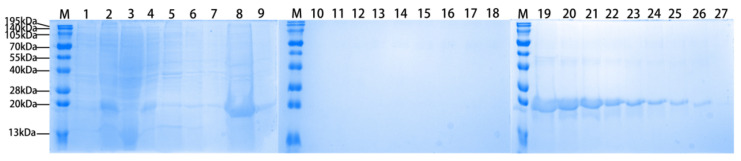
The induction expression and purification of recombinant Tarim red deer HGF/SF Receptor (MET) agonist protein. M: Servicebio Prestained Protein Marker VII (8–195 kDa); Lane 1: Total protein of uninduced bacterial cells; Lane 2: Total protein of bacterial cells induced at 18 °C for 24 h; Lane 3: Supernatant protein after sonication of bacterial cells; Lane 4: Precipitated protein after sonication of bacterial cells; Lane 5: Soluble protein washed with 50 mM Tris (pH 8.5), 500 mM NaCl, 0.4% Triton X-100; Lane 6: Soluble protein washed with 50 mM Tris (pH 8.5), 500 mM NaCl, 0.025% NP40; Lane 7: Soluble protein washed with 50 mM Tris (pH 8.5), 500 mM NaCl; Lane 8: Precipitated protein after washing; Lane 9: Soluble protein after refolding with Tris buffered 2 M arginine solution for 3 days; Lane 10: Diluted protein refolding supernatant with Tris-HCl 100 times; Lane 11–17: Wash buffer permeate. Lanes 18–27: Elution buffer elute target protein.

The protein samples during expression and purification were analyzed by SDS-PAGE, as shown in Figure 3.

### 3.2. The Recombinant Agonist Treatment Can Alleviate Liver Injury in ALD Mice

**Figure 4:**
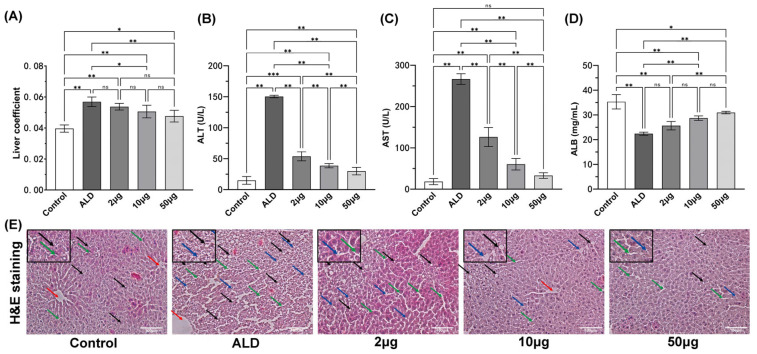
(**A**) Liver coefficient. Serum levels of ALT (**B**), AST (**C**), and ALB (**D**). (**E**) Liver H&E staining (200×) after treatment. The notation ‘ns’ indicates *p* > 0.05, signifying no significant difference, * *p* < 0.05, ** *p* < 0.01, Scale bar: 100 μm. Note: The black arrow shows steatosis vacuoles of hepatocytes, the green arrow shows bile ducts around hepatocytes, the red arrow shows central vein, and the blue arrow shows scattered lymphocyte infiltration, Scale bar: 100 μm.

The liver coefficient, defined as the ratio of liver weight to body weight, in the ALD model group of mice was significantly higher than that observed in the healthy control group (*p* < 0.01). Additionally, it was significantly elevated compared to the mice treated with 10 μg of recombinant protein (*p* < 0.05) and those treated with 50 μg of recombinant protein (*p* < 0.01). However, no significant differences were found between the various doses of recombinant protein treatment groups (*p* > 0.05, see Figure 4A).

In comparison to the control group, serum levels of ALT and AST were significantly elevated (*p* < 0.01), while ALB levels were significantly reduced (*p* < 0.01) in the ALD group. Treatment with 2 μg of recombinant protein resulted in a significant reduction in serum ALT and AST levels compared to the ALD group (*p* < 0.01, Figure 4B,C), with the reduction being dose-dependent. Additionally, the treatment groups receiving recombinant protein at doses of 10 μg and 50 μg exhibited a significant increase in serum ALB levels compared to the ALD model group (*p* < 0.01, Figure 4D), also demonstrating a dose-dependent relationship.

Representative histological findings of liver tissue, as illustrated in Figure 4E, revealed that the healthy control group of mice displayed intact and well-defined liver lobule structures, with no significant infiltration of inflammatory cells. The liver cells were well organized, featuring centralized and round nuclei. Conversely, the ALD model group exhibited considerable inflammatory cell infiltration surrounding the central vein, accompanied by disorganized liver cell arrangement and hepatocyte enlargement. In comparison to the ALD model group, the treatment groups receiving red deer recombinant protein (at doses of 10 μg and 50 μg) demonstrated significantly reduced edema and fibrotic inflammatory cell infiltration. Furthermore, the degree of bile duct dilation around the liver cells was diminished, the arrangement of liver cells improved, and these effects were positively correlated with the dose of recombinant protein administered.

### 3.3. The Recombinant Agonist Treatment Can Alleviate Liver Oxidative Stress in ALD Mice

**Figure 5:**
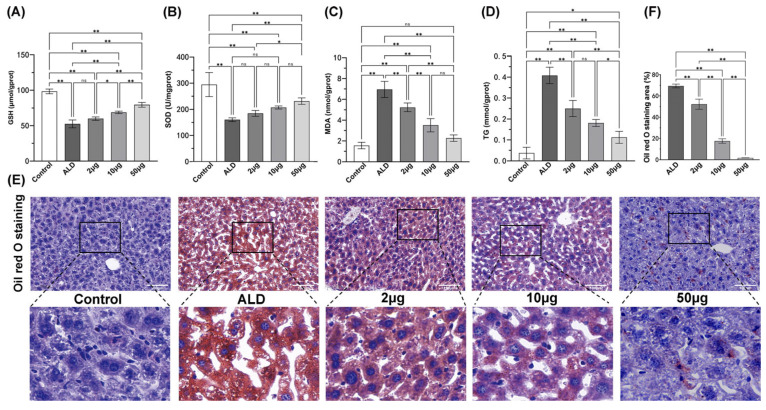
Levels of GSH (**A**), SOD (**B**), MDA (**C**) and TG (**D**) in the liver. (**E**) Liver oil red O staining (200×) after treatment. (**F**) Staining area of Oil Red O in Figure 4E. The notation ‘ns’ indicates *p* > 0.05, signifying no significant difference, * *p* < 0.05, ** *p* < 0.01, Scale bar: 100 μm.

The levels of GSH, SOD, and MDA in the supernatant of liver homogenate were assessed to evaluate oxidative stress in the liver. Compared to the control group, the ALD group exhibited a significant decrease in GSH and SOD levels (*p* < 0.01, Figure 5A,B), while MDA levels were significantly increased (*p* < 0.01, Figure 5C). Treatment with recombinant protein at doses of 10 μg and 50 μg resulted in a significant increase in GSH levels in the liver compared to the ALD group (*p* < 0.01, Figure 5A), with a dose-dependent improvement observed. Furthermore, treatment with 50 μg of recombinant protein significantly elevated SOD levels in the liver compared to the ALD group (*p* < 0.01, Figure 5B). Additionally, MDA levels in the liver were significantly reduced following recombinant protein treatment compared to the ALD model group (*p* < 0.01, Figure 5C), with a dose-dependent reduction also noted.

### 3.4. The Recombinant Agonist Treatment Can Reduce Liver Fat Deposition in ALD Mice

The results of liver TG detection indicated that the TG content in the liver of ALD mice was significantly higher than that in the healthy control group (*p* < 0.01). Following treatment with recombinant protein, the TG content in the liver of the treatment group was significantly reduced compared to the ALD model group (*p* < 0.01, Figure 5D).

The results of Oil Red O staining of liver tissue (Figure 5E) indicated that the liver tissue of healthy control group mice exhibited a lighter orange–red coloration, whereas the liver tissue of ALD mice displayed a darker orange–red hue. Notably, the liver tissue staining in the treatment group was significantly lighter compared to that of the ALD model group (*p* < 0.01, Figure 5F), with staining intensity decreasing as the doses of recombinant protein increased. These findings suggest that recombinant deer protein mitigates lipid metabolism disorders in mice with alcohol-induced liver injury in a dose-dependent manner, thereby reducing hepatic steatosis.

### 3.5. The Recombinant Agonist Treatment Can Promote Liver Cell Proliferation in ALD Mice

**Figure 6:**
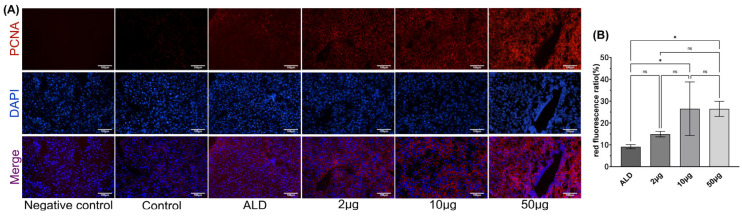
(**A**) Immunofluorescence of PCNA (200×) in liver after treatment. (**B**) Red fluorescence ratio. The notation ‘ns’ indicates *p* > 0.05, signifying no significant difference, * *p* < 0.05. Scale bar: 100 μm.

In the liver of ALD mice treated with recombinant Tarim red deer HGF via tail vein injection, an increase in PCNA labeling was observed (Figure 6A), This increase was positively correlated with the injection dosage of the recombinant protein administered (*p* < 0.05, Figure 6B).

## 4. Discussion

The HGF/c-Met signaling pathway is essential for organogenesis, embryogenesis, tissue and organ repair, hematopoietic cell differentiation, as well as the formation of the vascular and nervous systems, and bone remodeling [15,16]. Research indicates that within five minutes post-liver resection surgery, the tyrosine residues of c-Met begin to undergo phosphorylation, with levels gradually peaking at sixty minutes. Additionally, the concentration of HGF in the plasma increases 17-fold at 120 min following liver resection surgery [17,18]. Furthermore, c-Met deficiency results in liver necrosis and jaundice in rodent models, impairs regenerative capacity, and can lead to mortality [7]. Given its capacity to induce liver cell proliferation and promote liver regeneration, HGF presents a promising therapeutic agent for ALD.

The expression levels of biochemical markers in the blood or liver are crucial for assessing liver function impairment. Under normal conditions, the levels of various biochemical markers remain within a defined range. Following alcohol consumption, liver damage leads to significant alterations in the content or expression levels of these markers. For instance, when over 1% of liver cells experience necrosis, the serum alanine aminotransferase (ALT) level can increase by more than twofold [19]. Our data indicate that treatment with exogenous recombinant Tarim red deer HGF can effectively reduce ALT and AST levels, elevate ALB levels, and mitigate liver damage induced by ethanol metabolism.

Ethanol degradation in the body generates reactive oxygen species and various free radicals, including superoxide, peroxynitrite, hydrogen peroxide, and hydroxyl radicals. These species collectively promote glutathione depletion, free radical-mediated toxicity, lipid peroxidation, and immune responses through the production of pro-inflammatory cytokines, culminating in oxidative stress [20]. Oxidative stress is a principal pathological event in the development of alcoholic fatty liver disease, establishing a connection between lipid metabolism dysfunction and subsequent inflammation and cell apoptosis. Antioxidants such as SOD, GSH, and glutathione peroxidase (GSH-Px) in the liver mitigate peroxides and hydroxyl radicals within cells, thereby. preventing the peroxidation of unsaturated fatty acids in cell membranes and lowering the levels of MDA [21,22,23]. Furthermore, therapy with recombinant Tarim red deer HGF has been shown to successfully enhance the liver’s antioxidant stress response.

After alcohol consumption, lipid metabolism and synthesis pathways are affected, leading to abnormal accumulation of liver fatty acids and TG. Accumulating evidence suggests that HGF can significantly reduce lipid levels; Tomita et al.’s study showed that HGF treatment can downregulate the expression of SREBP-1c and SCD-2 in primary hepatocytes [24]. Jing et al. further found that HGF can significantly reduce the expression levels of SREBP-1c and FAS in high-fat diet-fed mice, and increase the expression of PPARα to prevent hepatic steatosis [25]. The use of the Met receptor agonist META4 can also improve liver function in mice with non-alcoholic fatty liver disease [26]. However, the exact mechanism by which the HGF/c-Met signaling pathway affects lipid synthesis is still not fully understood. In this experiment, the exogenous recombinant Tarim red deer HGF also reduced liver TG content and fat deposition.

Although this study did not directly measure the phosphorylation levels of c-Met or the activation of downstream signaling pathways, numerous studies have reported that HGF promotes hepatocyte proliferation and liver regeneration by activating the c-Met receptor, which subsequently activates downstream signaling pathways, including JAK/STAT3 and PI3K/Akt/NFκB [7,27]. For instance, it has been demonstrated that the intravenous injection of exogenous HGF into normal rats can induce hepatocyte proliferation and increase liver volume [28]. Moreover, research conducted by Kaibori et al. has shown that the administration of recombinant human HGF activator via the portal vein significantly enhances the rate of liver regeneration, with notable activation of the JAK/STAT3 and PI3K/Akt pathways in response to HGF stimulation [29]. Additionally, the study by Li et al. further confirmed that recombinant human HGF can inhibit inflammatory responses and improve liver function in mice with non-alcoholic fatty liver disease by promoting the phosphorylation of JAK2-STAT3 [10].

The HGF pathway interacts with the TGF-α pathway to directly stimulate DNA synthesis in liver cells, facilitating their transition from the G0 phase to the G1 phase [30]. PCNA is frequently employed as a marker for evaluating liver injury and repair, serving as an indicator of cell proliferation. It is primarily synthesized during the S phase of the cell cycle, and its biological function is intricately linked to cell proliferation and cycle regulation. Generally, the expression level and intensity of PCNA protein in cells can reflect their proliferative activity and is positively correlated with the extent of DNA synthesis and replication activity. Consequently, we assessed liver cell proliferation by detecting PCNA expression through immunofluorescence in the liver. As anticipated, the administration of the exogenous recombinant protein effectively promoted liver cell proliferation, akin to the effects of HGF. Drawing from the existing literature and the significant therapeutic effects of the recombinant Tarim red deer HGF, it is reasonable to speculate that its mechanism of action is closely associated with the activation of the HGF/c-Met signaling pathway and subsequent downstream signal transduction.

Combining blood and liver biochemical indicators with liver tissue sections, we confirmed that recombinant Tarim red deer HGF significantly alleviates ethanol-induced liver injury in vivo, enhances the antioxidant capacity of the liver in mice with alcoholic liver disease, reduces hepatic lipid deposition, and promotes hepatocyte proliferation. These short-term results indicate that recombinant Tarim red deer HGF exhibits promising initial therapeutic effects, providing a robust foundation for further investigation into its long-term effects. For instance, the study by Vallarola et al. [31] demonstrated that administering 25 μg of recombinant HGF-K1K1 protein to mice daily for 5 consecutive days significantly alleviated symptoms in mouse models of amyotrophic lateral sclerosis without noticeable toxic reactions. Similarly, Nola et al. [11] showed that injecting mice with 10 μg of recombinant human HGF-K1K1 protein per dose, for a total of five injections, effectively treated mouse models of fatty hepatitis. These findings further support the safety and efficacy of recombinant HGF proteins, providing valuable references for the long-term studies in our research. Interestingly, in vivo experiments indicated that the effects of recombinant Tarim red deer HGF are dose-dependent.

## 5. Conclusions

Our study demonstrated that recombinant HGF derived from Tarim red deer significantly enhanced the liver’s antioxidant capacity, improved lipid deposition in the liver, and promoted hepatocyte regeneration, thereby effectively reducing liver damage in a mouse model of alcoholic liver disease.

## Figures and Tables

**Table 1:** Homologous recombination fragment primers.

	Primer Sequence	Length
Fragment 1	F: AGAAGGAGATATACCATGGCCATTAGAAACTGTATCATTGGGAAA	296 bp
	R: CCTTTCCCAATGATGCATTCAACTTCTGAA	
Fragment 2	F: AAGTTGAATGCATCATTGGGAAAGGCGGT	301 bp
	R: CCAGACTCGAGTGCGGCCGCTCATCAATGATGATGATGATGATGTTCAACTTCTGAACACTGAGGAATG	
Liner vector	F: GGCCATGGTATATCTCCTTCTT	5152 bp
	R: GCGGCCGCACTCGAGTCT	

## Data Availability

The original contributions presented in the study are included in the article; further inquiries can be directed to the corresponding authors.
